# DNA methylation assessment as a prognostic factor in invasive breast cancer using methylation-specific multiplex ligation dependent probe amplification

**DOI:** 10.17179/excli2015-485

**Published:** 2016-01-11

**Authors:** Halaleh Shakeri, Jalal Gharesouran, Ashraf Fakhrjou, Ali Esfahani, Seyyed Mojtaba Mohaddes Ardebili

**Affiliations:** 1 Hematology & Oncology Research Center, Tabriz University of Medical Sciences, Tabriz, Iran; 2 Department of Biochemistry and Clinical Laboratories, Division of Medical Genetics, Faculty of Medicine, Tabriz University of Medical Sciences, Tabriz, Iran; 3 Department of Pathology, School of Medicine, Tabriz University of Medical Sciences, Tabriz, Iran

**Keywords:** MS-MLPA, methylation, breast cancer, IDC, tumor suppressor genes

## Abstract

DNA methylation of promoter regions is a common molecular mechanism for inactivation of tumor suppressor genes that participates in carcinogenesis. Determining the methylation status of genes in cancer and their association with clinical features play an essential role in early diagnosis, prognosis and determine appropriate treatment for patients. The purpose of the present study was to evaluate the methylation of tumor suppressor genes in patients with invasive ductal carcinoma (IDC). Furthermore, we evaluated the association between clinical parameters and DNA methylation as a biomarker in diagnostic IDC patients. The methylation-specific multiplex ligation dependent probe amplification (MS-MLPA) assay was used to analyze the methylation profile of 24 genes in formalin-fixed paraffin embedded (FFPE) tissue samples from 75 patients with IDC. Each of the patients showed a distinctive methylation profile. We observed higher methylation in the RASSF1 (48 %), CDH13 (44 %) and GSTP1 (36 %) genes. Some of the methylated genes were associated with clinical features. Methylation of GSTP1 (P=0.028) and RASSF1 (P=0.012) were related with lymph node metastasis. Methylation of GSTP1 (P=0.005) was associated with high histological grade. Moreover, concurrent methylation of GSTP1 and CDH13 was observed in IDC patients (p<0.001). Hierarchical cluster analysis based on the methylation profile revealed two main clusters of patients, the highly methylated cluster being significantly associated with high histological grade and lymph node metastasis. The results of this study indicate that the methylation status of RASSF1 and CDH13 and GSTP1 can be a prognostic marker to better management of IDC patients.

## Introduction

Breast cancer is a heterogeneous disease and the common tumor among women worldwide (Vo and Millis, 2012[39]). Invasive breast carcinoma has been categorized by morphological criteria into invasive ductal carcinoma (IDC), invasive lobular carcinoma (ILC), and other less common subtypes. IDC is the most common type and comprises about 80 % of the malignant form. ILC is the second most common type of invasive breast cancer after IDC. This type accounts for about 8-14 % (Hoque et al., 2009[17]).

Epigenetic alterations are the primary changes in cancer progression and play important roles in the pathogenesis of cancer. One of the most common epigenetic changes in cancers is methylation of CPG islands in gene promoter regions. Such changes contribute to the process of tumorigenesis by silencing tumor suppressor genes (Delpu et al., 2013[9]; Hatzimichael et al., 2014[16]; Barrow and Michels, 2014[2]). These genes play significant roles in the regulation of cell cycle, DNA repair, apoptosis and signal transduction (Esteller et al., 2001[11]).

Clinicopathological features routinely are used as strong predictive factors in the evaluation of breast cancer. These features include lymph node metastasis, histological grade and tumor size that indicate the malignant potential of tumors (Rakha et al., 2008[28]). Lymph node metastasis correlates with the risk of distant recurrence in patients. There is the most consistent prognostic factor used in adjuvant therapy decision making. For patients with non-metastatic lymph nodes, tumor size is a predictive marker. Tumor grade does have significance for patients with non-metastatic lymph node and borderline tumor sizes (Cianfrocca and Goldstein, 2004[6]).

Recent studies indicate that there are associations between methylation in some genes and clinicopathological parameters in breast cancer. Methylation in CDH13 is associated with tumor size (Xu et al., 2012[42]). GSTP1 methylation is associated with tumor size and nodal metastasis (Arai et al., 2006[1]). Furthermore, an association of RARB promoter methylation with lymph node metastasis has been reported (Marzese et al., 2012[22]). The identification of methylated genes and their relationship to clinical features can contribute to the prognosis and early detection of tumor. 

In recent years, the role of aberrant methylation in the development of invasive breast cancer has been the subject of various researches yielding controversial outcomes (Parrella et al., 2004[27]; Lewis et al., 2005[20]). Previous studies have pointed that methylation profiles are specific for each type of human cancer and different in various ethnicities (Esteller et al., 2001[11]). In spite of intensive studies, the pathogenesis of IDC has not been completely revealed (Parrella et al., 2004[27]; Lewis et al., 2005[20]). A recent study has pointed to the presence of a unique methylation profile in IDC (Marzese et al., 2012[22]). However, this profile and its relation to clinical features are not clear yet (Xu et al., 2012[42]; Marzese et al., 2012[22]) because most of previous studies have investigated methylation in only a few genes for pure IDC cases (Ramalho et al., 2014[29]; Mirza et al., 2007[23]).

Several techniques exist to assess gene methylation status. However, most of them are able to examine only one gene at a time (Pang et al., 2013[26]). The MS-MLPA is a new, relatively simple and sensitive technique for detecting the methylation status of multiple genes in a single reaction. This method can be used to determine the profile of methylated genes in different types of cancer (Nygren et al., 2005[25]).

The purpose of this research was improvement of our understanding of promoter methylation of tumor suppressor genes in IDC samples using MS-MLPA assay. Furthermore, we evaluated the association between clinical parameters and methylation of tumor suppressor genes in order to a better understanding of the pathogenesis and heterogeneity of breast carcinoma.

## Materials and Methods

### Patients and tissue samples

In this study, formalin-fixed paraffin embedded (FFPE) samples from 80 patients with breast carcinoma were acquired from the archive of the pathology laboratories in the Tabriz (Iran). Furthermore, 10 normal tissues were considered as control samples. Previously, written informed approval was acquired from all patients. All samples were reviewed by a pathologist to confirm the diagnosis, according to World Health Organization criteria. Tumors were histologically graded from 1 to 3 according to the Nottingham Histologic score (Elston and Ellis, 2002[10]). To evaluate a group of patients with similar clinical features, we selected only pure invasive ductal carcinomas (IDCs) (n=75) from the 80 obtained samples.

Pathological files were reviewed to record clinicopathological parameters such as age, grade, lymph node status and tumor size (Table 1(Tab. 1)). The mean age of these patients was 48 years (range 28 to 68 years). The samples of normal breast were retrieved from patients that had undergone surgery for diagnosis. Breast cancer in these samples was excluded by the pathologist. Use of FFPE samples for this research was approved by the ethics boards of the Hematology and Oncology Research Center, Tabriz University of Medical Sciences.

### DNA extraction

Three to four sections (5-8 µm thick) were acquired from each paraffin block. To remove paraffin, the samples were incubated at 75 °C for 15 minutes and washed with Xylene (3×10 minutes). DNA was extracted using QIAamp DNA FFPE kit (Qiagen) according to the manufacturer's guidelines. The quantity and purity of DNA samples were evaluated by NanoDrop 1000 spectrophotometer (Thermo Scientific, DE, USA). 

### MS-MLPA assay

DNA methylation was assessed by MS-MLPA using the ME001-C2 tumor suppressor kit (MRC-Holland, Amsterdam, and The Netherlands). The ME001-C2 kit contains 26 different probes for 24 tumor suppressor genes. These genes are frequently inactivated by methylation in tumors, but are unmethylated in healthy subjects. In addition, the kit includes 15 reference probes, which remain uncut after digestion by HhaI restriction enzyme. Information about the length of the probes, genes locus and sequence with HhaI site can be found at MRC-Holland website.

The MS-MLPA method is based on the probes to determine methylation quantification. The probes bind to the target sequences which contain a cleavage location for the methylation-sensitive HhaI enzyme. Procedures were performed according to the kit manufacturer's instructions with slight modification. After DNA denaturation and probe hybridization, each of the samples is divided into two microtubes. One of them is incubated with the HhaI enzyme. If the sample DNA is unmethylated, hybrids of probe and DNA will be digested by HhaI and will not generate a signal in capillary electrophoresis analysis. However, methylated DNA is prevented from being digested by enzyme. As a result, the ligated probes will be amplified by PCR and the signal will be generated.

PCR products were separated by the capillary electrophoresis sequencer (ABI 310; Applied Biosystems, Foster City, CA, USA) and the results were analyzed by GeneMarker software (version 2.6, Soft Genetics, State College, PA). Based on previous studies, a methylation of gene was considered as positive when the methylation ratio was higher than 0.15 (Moelans et al., 2011[24]).

### Statistical analysis

The K2 test was used to examine the relations between methylation status of CPG islands and clinicopathological variables (age, grade, tumor size and lymph node metastasis). Associations with P-value <0.05 were considered as significant. Statistical analyses were performed using SPSS version 22 software. Furthermore, hierarchical clustering with Manhattan distance and complete linkage was performed using MATLAB (R2013b) software.

## Results

### Methylation status by MS-MLPA

Analysis of tumor suppressor genes using MS-MLPA, indicated that all 24 genes were unmethylated in the normal samples (n = 10) while, methylation patterns in all the IDC samples were manifestly variable. Each of the patients showed a distinctive methylation profile. In 21 genes, methylation was observed in at least one tumor tissue.

**Figure 1:**
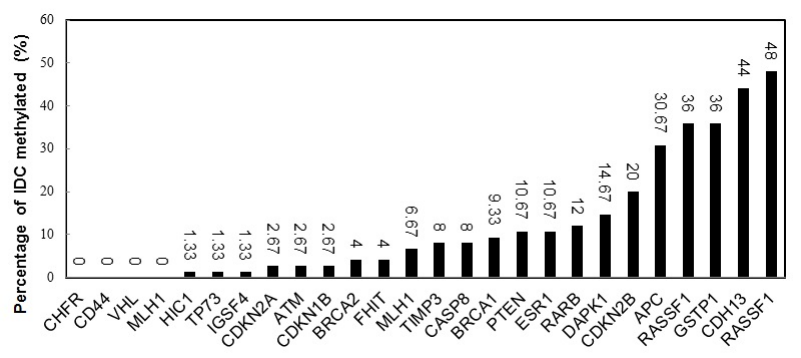
Frequency distribution of DNA methylation for each of 26 analyzed CpG islands among 75 IDCs

**Figure 2:**
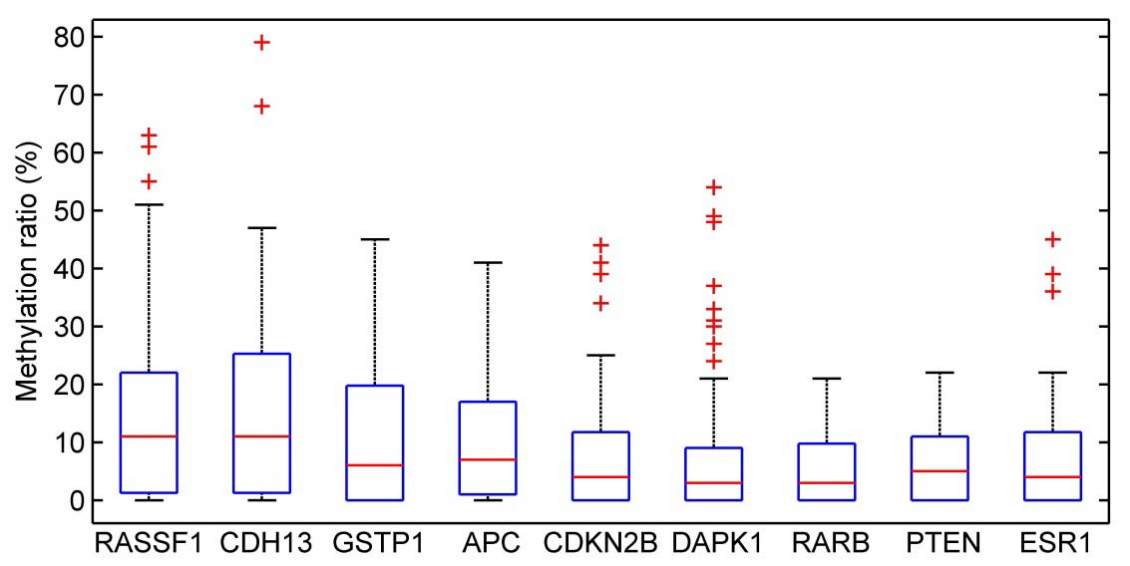
Methylation levels of the nine significant genes showed as box plotAssociation between DNA methylation and clinicopathological parameters

The most frequently methylated genes were RASSF1 (48 %), CDH13 (44 %) and GSTP1 (36 %). Methylation was not observed in MLH1, VHL, CD44 and CHFR gene promoters in IDC patients. However, in two tumor samples, 9 genes were detected as methylated. Frequency distribution of DNA methylation for each of 26 analyzed genes is displayed in (Figure 1(Fig. 1)) and to better understand how values are spaced, methylation levels of the nine significant genes are shown as box plot (Figure 2(Fig. 2)). The ME001-C2 kit contains two different CpG islands for RASSF1 gene (328 bp and 382 bp). One of them was methylated in 48 % of IDC patients and the other in 36 %.

### Association between DNA methylation and clinicopathological parameters

In the current study, we investigated the association between three genes with most frequently methylation (RASSF1, CDH13 and GSTP1) and clinicopathological parameters of the patients. Furthermore, we analyzed IDC patients for concurrent methylation of these genes.

**Figure 3:**
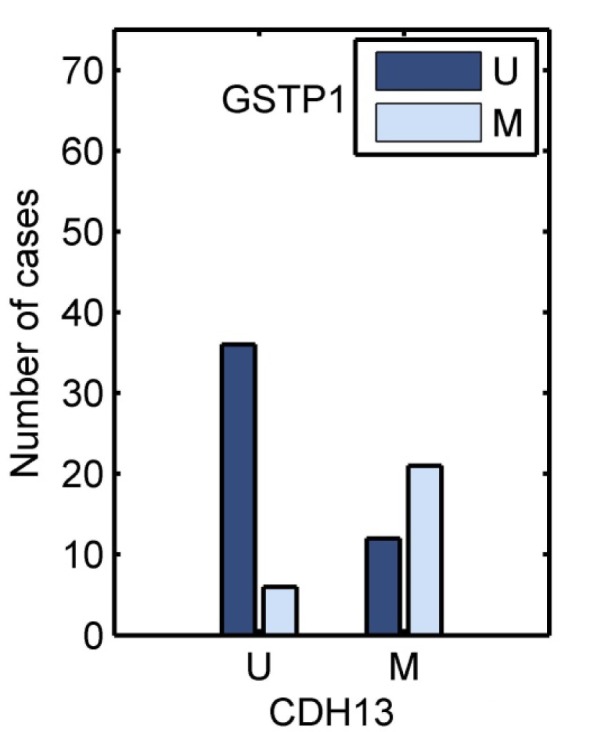
Concurrent methylation of CDH13 and GSTP1 in IDC patients (P < 0.001)

Methylation of RASSF1 (P= 0.012) and GSTP1 (P=0.028) were associated with lymph node metastasis. Methylation of GSTP1 (P=0.005) showed a statistically significant association with high histological grade. However, no statistically considerable association was found between CDH13 methylation and clinicopathological features. In addition, a significant correlation between patient's age and tumor size with methylation status was not observed (Table 2(Tab. 2)). Moreover, methylation status of GSTP1 was associated with methylation of CDH13 (Figure 3(Fig. 3)) but, no statistically considerable association was found between methylation of other genes.

### Cluster analysis

**Figure 4:**
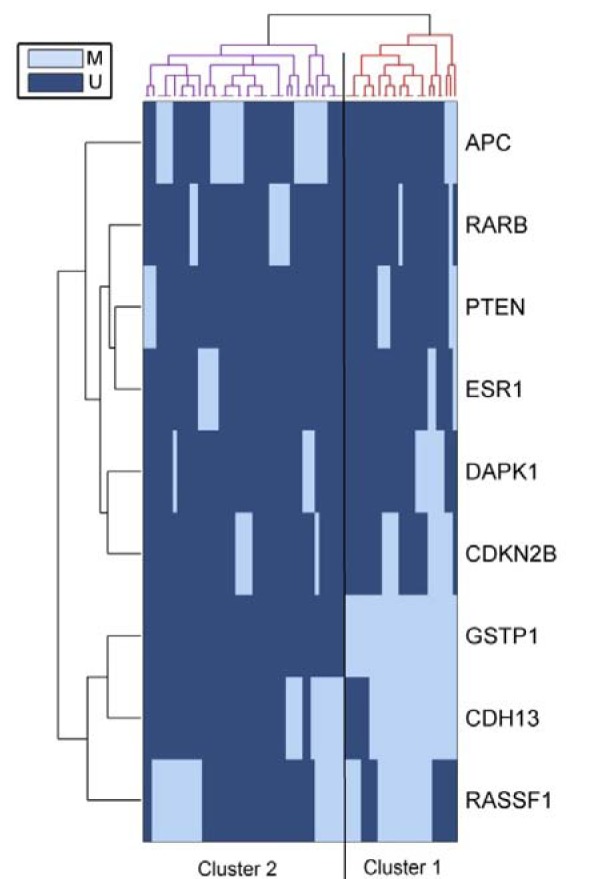
Unsupervised hierarchical cluster analysis of the methylation profile of 75 IDCs using the information from 9 genes.

Unsupervised hierarchical clustering was applied to classify cases with correlated methylation profiles and genes with correlated methylation patterns. For this analysis, to decrease the effect of genes that were rarely or never methylated, we selected genes that methylated in more than 10 % of the IDC patients. This analysis based on the methylation profile revealed two main clusters of patients; cluster 1 samples were characterized by RASSF1A, CDH13 and GSTP1 methylation but cluster 2 samples showed APC and RASSF1 methylation (Figure 4(Fig. 4)). Cluster 1 being significantly associated with high histological grade (P=0.005) and lymph node metastasis (P=0.028) compared with cluster 2.

## Discussion

IDC is the most common type of malignant breast cancer. Identification of the molecular biological mechanisms in IDC contributes to a well understanding of the disease, early diagnosis and determining the appropriate treatment strategies. Aberrant DNA methylation is an important epigenetic alteration that occurs in the early stages of human tumors, including breast cancer (Brooks et al., 2009[4]).

The objective of the present study was to assess the methylation in promoter regions of 24 tumor suppressor genes on samples prepared from 75 IDC subjects and 10 controls using MS-MLPA. In addition, we investigated for any associations between the methylation status of gene promoters and the clinicopathological features for higher methylated genes.

The most frequently methylated genes in our study were RASSF1, CDH13 and GSTP1. However, methylation was not observed in MLH1, VHL, CD44 and CHFR genes. Previous studies have demonstrated that RASFF1 and APC are the most frequently methylated genes in IDC (Marzese et al., 2012[22]).

RASSF1 is a tumor suppressor gene that is involved in apoptotic pathways, signal transduction and regulates cell proliferation. Methylation of RASSF1 has been commonly observed in a variety of human tumor types (Wei et al., 2013[40]; Dammann et al., 2005[7]).

The highest frequency of RASSF1 methylation (70 % and 71.4 %) has been reported in IDC patients from India and Argentina (Marzese et al., 2012[22]; Brooks et al., 2009[4]). The methylation frequency of RASSF1 gene (48 %) obtained in our study is close to the results previously reported by other researchers for Iranian IDC patients (50 %) (Rasti et al., 2009[30]). Possible reasons behind these differences can be included ethnic diversity and tumor tissue heterogeneity.

Many studies have reported that the most methylated gene in breast cancer is RASSF1 (Brooks et al., 2009[4]; Cho et al., 2012[5]). Although it may be silenced by deletion or point mutations, promoter hypermethylation is a common mechanism in loss of function of RASSF1 in cancer (Yoon et al., 2001[43]). Therefore, this can be a reason for the higher-frequency of methylation in RASSF1 relative to other genes in breast tumors.

In this study methylation of RASSF1 showed a statistically significant relationship with lymph node metastasis that has been reported by previous studies (Dammann et al., 2005[7]). This result seems to be a useful prognostic biomarker in breast cancer.

CDH13 (H-Catherine) was the second most methylated gene in our results (44 % of cases). CDH13 is a cell adhesion protein and decreased expression of this gene plays an essential role in tumor metastasis. Methylation in CHD13 has been reported in several cancers including invasive bladder, ovarian and breast cancers (Lin et al., 2014[21]; Bol et al., 2010[3]). It has been found to be methylated in 33 % of breast cancer patients (Xiang et al., 2013[41]). But, CDH13 methylation has not been extensively examined in IDC. Xu et al. (2012[42]) indicated that CDH13 methylation in breast cancer was associated with tumor size, while in our study no statistically association was found between CDH13 methylation and clinicopathological features.

GSTP1 was the third most frequently methylated gene (36 % of cases). The methylation rate of GSTP1 remains controversial in IDCs because of the variation of reported frequencies ranging from 0 to 39 % (Tserga et al., 2012[38]).

Previous studies indicated that GSTP1 methylation is more likely a late event in the pathogenesis of breast tumors (Arai et al., 2006[1]). Recently, GSTP1 methylation was shown to be significantly associated with increased age, tumor size and lymph node metastasis (Arai et al., 2006[1]; Xiang et al., 2013[41]; Shinozaki et al., 2005[34]). In this study, GSTP1 methylation exhibited a trend toward relationship with lymph node metastasis and increasing IDC grade. 

A recent study has shown concurrent methylation of GSTP1 and RASSF1 in IDC patients (Sharma et al., 2009[33]), while in our study no statistically association was found between these genes. Only, GSTP1 methylation was signiﬁcantly associated with CDH13 methylation. This association suggests that these genes do not appear to be methylated alone. Some tumors exhibit concurrent methylation of several genes, a phenomenon known as the CpG island methylator phenotype (CIMP), which is tumor type specific. Moreover, in invasive breast cancers, a breast CIMP (B-CIMP) has been described and associated with clinical outcome. However the molecular mechanisms generating the concurrent methylation are still unknown (Fang et al., 2011[12]). But, there is a hypothesis that overexpression of DNA methyltransferase (DNMT) causes methylation of specific clusters of genes in breast cancer (Giordano and Normanno, 2009[15]).

Hierarchical cluster analysis based on the methylation profile revealed two main clusters of patients, the highly methylated cluster being significantly associated with high histological grade and lymph node metastasis. Our results demonstrated that methylation statuses of genes could be used to classify IDC patients into the same groups and in consequence they receive the same treatment.

In order to compare MS-MLPA with other methods, some of earlier studies were investigated. Several studies using methylation-speciﬁc polymerase chain reaction (MSP) method have demonstrated methylation of RASSF1A (51 %) (Rasti et al., 2009[30]), GSTP1 (34.4 %) (Saxena et al., 2012[32]), APC (36 %) (Jin et al., 2001[18]) and CDH13 (33 %) (Toyooka et al., 2001[36]) in breast cancer samples. Furthermore, Feng and colleagues (2007[13]) analyzed the methylation of multiple genes in breast cancer using bisulfite pyrosequencing and found that RASSF1 was methylated in 58 % of breast tumors, CDH13 in 44 % and RARB in 17 %. Also, APC and RASSF1, using quantitative MSP method, have been found to be methylated in 42 % and 48.6 % of breast cancer samples, respectively (Lee et al., 2004[19]; Stuopelyte et al., 2013[35]). These results are almost similar to the findings of our study that show the concordance between MS-MLPA and other methods to assess gene methylation status. Also, several studies have compared MS-MLPA with MSP or pyrosequencing and showed a good concordance between MS-MLPA and these methods (Furlan et al., 2013[14]). Roessler and colleagues (2015[31]) recently studied genome-wide DNA methylation patterns for breast cancer subtypes using the Infinium Human Methylation 450k (HM450k) BeadChip. In general, this method is a proper tool to perform large-scale DNA methylation profiling. But, HM450k analysis and interpretation are more complex than initially thought (Dedeurwaerder et al., 2014[8]). Moreover, no standardized statistical guideline for the evaluation of HM450K BeadChip data has been established (Roessler et al., 2015[31]). But MS-MLPA is a relatively simple and sensitive technique for detecting the methylation status (Nygren et al., 2005[25]). Also, Trabelsi and colleagues (2015[37]) have demonstrated a concordance between MS-MLPA and HM450k BeadChip.

Comparison of the results obtained from the present study with previous reports showed that the methylation statuses of genes and reported frequencies are highly variable. These may be due to differences in the populations and ethnicities that were studied. More reasons for this variation are differences in sample size, methylation assessment techniques and statistical methods in each study.

In present study no follow-up data about the IDC patients are available to define whether DNA methylation is related with outcome in IDC patients. Therefore, this relationship should be validated in further researches in large groups of patients with follow-up to improve invasive breast carcinoma management. 

## Conclusion

The present findings indicate that methylation of RASSF1, CDH13 and GSTP1 can be used as prognostic factors to better management of IDC patients. However, these findings should be approved by further studies prior to use as epigenetic markers on IDC. 

## Acknowledgements

Authors would like to thank Hematology and Oncology Research Center, Tabriz University of Medical Sciences for supporting this project (grant number: 92.13) which was a part of MSc thesis (92/2-1/4).

## Conflict of interest

The authors have no conflict of interest.

## Figures and Tables

**Table 1:**
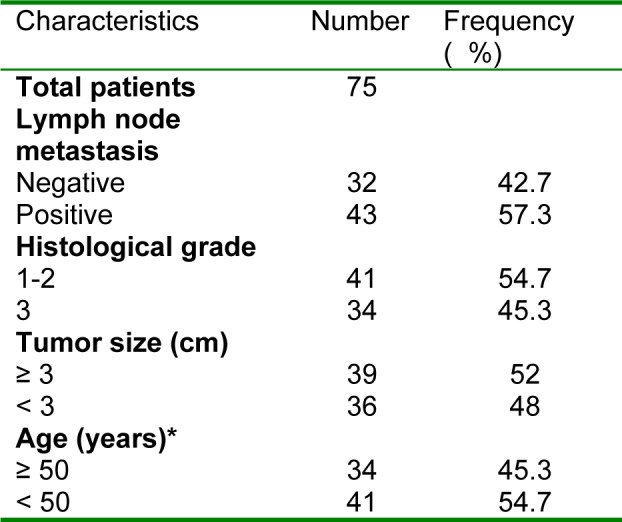
Clinical and pathological characteristics of samples with IDC (N=75)

**Table 2:**
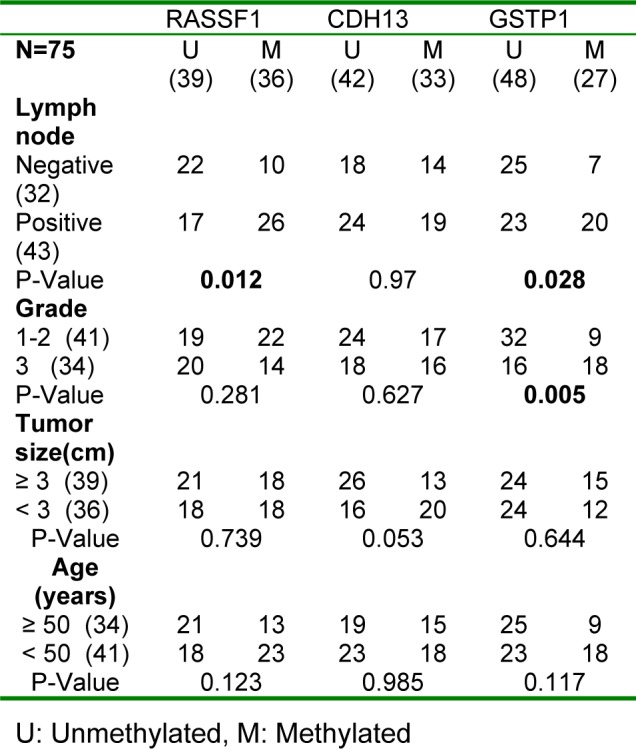
Association between the methylation status and the clinicopathological characteristics of IDC patients
